# Immunohistochemical evaluation of myofibroblast density in odontogenic cysts and tumors

**DOI:** 10.15171/joddd.2016.006

**Published:** 2016-03-16

**Authors:** Maryam Kouhsoltani, Monireh Halimi, Golchin Jabbari

**Affiliations:** ^1^Dental and Periodontal Research Center, Tabriz University of Medical Sciences, Tabriz, Iran; ^2^Assistant Professor, Department of Oral and Maxillofacial Pathology, Faculty of Dentistry, Tabriz University of Medical Sciences, Tabriz, Iran; ^3^Hematology Oncology Research Center, Tabriz University of Medical Sciences, Tabriz, Iran; ^4^Associate Professor, Department of Pathology, Faculty of Medicine, Tabriz University of Medical Sciences, Tabriz, Iran; ^5^Drug Applied Research Center, Tabriz University of Medical Sciences, Tabriz, Iran; ^6^Postgraduate Student, Department of Endodontics, Faculty of Dentistry, Tabriz University of Medical Sciences, Tabriz, Iran

**Keywords:** Myofibroblasts, odontogenic cysts, odontogenic tumors

## Abstract

***Background. ***The aim of this study was to investigate myofibroblast (MF) density in a broad spectrum of odontogenic cysts and tumors and the relation between the density of MFs and the clinical behavior of these lesions.

***Methods.*** A total of 105 cases of odontogenic lesions, including unicystic ameloblastoma (UAM), solid ameloblastoma (SA), odontogenic keratocyst (OKC), dentigerous cyst (DC), radicular cyst (RC) (15 for each category), and odontogenic myxoma (OM), adenomatoid odontogenic tumor (AOT), calcifying odontogenic cyst (COC) (10 for each category), were immunohistochemically stained with anti-α-smooth muscle actin antibody. The mean percentage of positive cells in 10 high-power fields was considered as MF density for each case.

***Results. ***A statistically significant difference was observed in the mean scores between the study groups (P < 0.001). The intensity of MFs was significantly higher in odontogenic tumors compared to odontogenic cysts (P < 0.001). There was no statistically significant difference between odontogenic tumors, except between UAM and OM (P = 0.041). The difference between OKC and odontogenic tumors was not statistically significant (P > 0.05). The number of MFs was significantly higher in OKC and lower in COC compared to other odontogenic cysts (P = 0.007 and P = 0.045, respectively).

***Conclusion.*** The results of the present study suggest a role for MFs in the aggressive behavior of odontogenic lesions. MFs may represent an important target of therapy, especially for aggressive odontogenic lesions. Our findings support the classification of OKC in the category of odontogenic tumors.

## Introduction

Odontogenic lesions are an important aspect of oral and maxillofacial pathology. These lesions arise from odontogenic apparatus (forming dental organ or related structures) and consist of two main categories: odontogenic cysts and odontogenic tumors.^1,2^

Odontogenic cysts and tumors, particularly those with aggressive behaviors (the lesions with infiltrative potentials and high growth and recurrence rates), are important due to the bone destruction they cause.^1-3^ The pathogenesis of odontogenic lesions is not clearly defined and different cell types may play roles in this process.^2,3^ It is now well-established that harmonized interactions between the epithelial and stromal cells are critical in controlling the growth and clinical behavior of pathoses.^4,5^ Myofibroblasts (MFs) are the important cellular components of the stroma. These cells, initially described in the granulation tissue, are fibroblasts that have been specialized by secretion of TGF-β1 and have developed structural features of smooth muscle cells, including the capability to express α-smooth muscle actin.^6,7^ Complex interactions exist between MFs and their microenvironment as well as the epithelial cells. These cells are able to secret the extracellular matrix and also remodel the extracellular matrix by secreting matrix metalloproteinases.^3-5,8^

Few studies have assessed the role of MFs in odontogenic cysts with controversial findings.^3,5,9^

In this study, our aim was to evaluate immunohistochemically the number of MFs in a wide spectrum of odontogenic lesions. In the case of contribution of MFs in more aggressive lesions, treatment strategies, including pharmacologic agents, can be used to inhibit MF activity as an aid to reduce mutilating surgeries (especially in cases of aggressive tumors). In the present study, anti-α-smooth muscle actin (α-SMA) antibody, a very useful immunohistochemical marker for identification of MFs,^10^ was used.

## Methods

The research protocol was approved by the Ethics Committee of Tabriz University of Medical Sciences (Ref. No. 450). In this cross-sectional study, 105 formalin-fixed,‏ paraffin-embedded tissue blocks of odontogenic lesions were retrieved from the archives of three laboratories at Tabriz University of Medical Sciences, Tabriz, Iran. The specimens included unicystic ameloblastoma (UAM), solid ameloblastoma (SAM), odontogenic keratocyst (OKC), dentigerous cyst (DC), radicular cyst (RC) (15 cases for each category), and odontogenic myxoma (OM), adenomatoid odontogenic tumor (AOT), calcifying odontogenic cyst (COC) (10 cases for each category). Only the cases with sufficient clinical and radiographic records were included and all hematoxylin-and-eosin stained slides were reviewed to confirm the diagnosis. Samples with inappropriate paraffin blocks or inadequate microscopic fields were excluded.

Formalin-fixed, paraffin-embedded tissue blocks were used to obtain sections measuring 4 μm in thickness. The sections were stained through standard immunohistochemical staining methods according to the manufacturer's instructions (DAKO, Glostrup, Denmark). The sections were mounted on glass slides, deparaffinized in xylene, rehydrated through graded alcohol and incubated in 1% hydrogen peroxide at room temperature to inhibit peroxidase activity. For antigen retrieval, the slides were placed in citrate-buffer solution (pH=6.0, 0.01 M) for 10 minutes. Subsequently, the sections were exposed to the primary antibody (anti-α-smooth muscle actin) for 30 minutes at room temperature and then rinsed with phosphate-buffered saline (PBS) and incubated with the secondary antibody. Reaction products were detected using 0.3% diaminobenzidine (Dako Cytomation) solution. Finally, the sections were counterstained with Harris hematoxylin.

For immunohistochemical analysis, 10 representative high-power fields (HPFs) were chosen for each sample. The percentage of positive cells (the cells with a clearly defined immunostaining) in each field was calculated. The mean percentage of 10 HPFs was considered as α-SMA expression for each case and classified as negative (score 0) when <5% of cells were positive, weak (score 1) when 5%–50% of cells were positive, or strong (score 2) when >50% of cells were positive.^11^

### Statistical analysis

Data were analyzed statistically using SPSS 20.0 (SPSS, Chicago, IL). The results were expressed as means ± standard deviations (SD). Kruskal-Wallis test was used to compare the MF density between the study groups and subsequently, Mann-Whitney U test was carried out to compare the scores between two independent groups. Significance was established at a P < 0.05.

## Results

**Figure 1:**
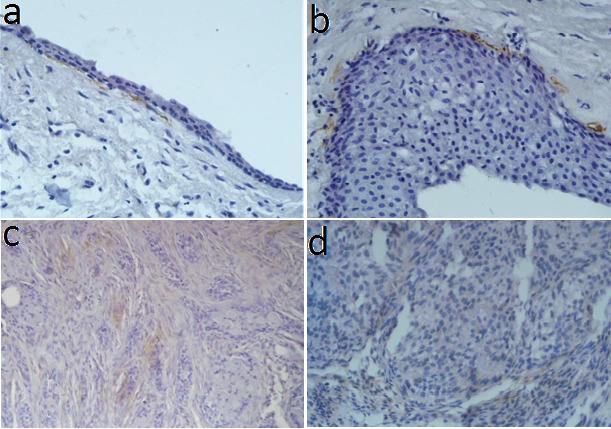
α-SMA-positive myofibroblasts in: a) dentigerous cyst, b) odontogenic keratocyst, c) solid ameloblastoma, d) ademonatoid odontogenic tumor (IHC stain, ×400).

α-SMA-positive spindle cells were mostly located beneath and parallel to the epithelium in odontogenic cysts. However, occasional α-SMA-positive aggregates were observed in the cyst wall. Layers of α-SMA + cells surrounded islands of odontogenic epithelium, as well as the periphery of the blood vessels, in odontogenic tumors (Figure 1).

### Odontogenic keratocyst

Of the 15 cases of odontogenic keratocyst, 2 (13.3%) did not express α-SMA; 9 (60%) exhibited weak expressions; and 4 (26.7%) revealed strong expressions. These findings represented a relatively high expression of α-SMA in odontogenic keratocysts.

### Dentigerous cyst

Of the 15 specimens of dentigerous cysts, 7 (46.7%) had no expression of α-SMA; 3 (20%) revealed weak expression; and 5 (33.3%) showed strong expressions. These findings indicated relatively low expression of α-SMA in dentigerous cysts.

### Radicular cyst

The expression of α-SMA was negative in six samples (40%); 8 cases (53.3%) showed weak expression; and 1 case (6.7%) showed strong expression. Therefore, a relatively low expression of α-SMA was seen in periapical cysts.

### Calcifying odontogenic cyst 

COC was the only lesion that demonstrated no strong expression of α-SMA; 2 cases (20%) represented weak expressions and 8 cases (80%) showed negative expressions, indicating a very low expression of α-SMA in COCs.

### Unicystic ameloblastoma

Three cases (20%) of unicystic ameloblastoma showed negative expression of α-SMA; 8 cases (53.3%) showed weak expression; and 4 cases (26.7%) showed strong expressions, representing moderate expression of α-SMA in unicystic ameloblastomas.

### Solid ameloblastoma

Of the 15 samples of solid ameloblastoma, 1 case (6.7%) showed no expression of α-SMA; 9 cases (60%) revealed weak expression; and 5 samples (33.3%) showed strong expression of α-SMA, indicating a relatively high expression of α-SMA in solid ameloblastomas.

**Table 1:** α-SMA expression in odontogenic cysts and tumors

**Groups**	**Negative α-SMA expression (number/percentage)**	**Weak α-SMA expression (number/percentage)**	**Strong α-SMA expression (number/percentage)**	**Number of cases**
**OKC**	2 (13.3%)	9 (60%)	4 (26.7%)	15
**DC**	7 (46.7%)	3 (20%)	5 (33.3%)	15
**RC**	6 (40%)	8 (53.3%)	1 (6.7%)	15
**UAM**	3 (20%)	8 (53.3%)	4 (26.7%)	15
**SAM**	1 (6.7%)	9 (60%)	5 (33.3%)	15
**AOT**	0 (0%)	6 (60%)	4 (40%)	10
**OM**	0 (0%)	3 (30%)	7 (70%)	10
**COC**	8 (80%)	2 (20%)	0 (0%)	10

OKC: odontogenic keratocyst; DC: dentigerous cyst; RC: radicular cyst; UAM: unicystic ameloblastoma; SAM: solid ameloblastoma; AOT: adenomatoid odontogenic tumor; OM: odontogenic myxoma; COC: calcifying odontogenic cyst.

### Adenomatoid odontogenic tumor 

Of 10 adenomatoid odontogenic tumors, no negative expression was observed; 6 cases (60%) demonstrated poor and 4 cases (40%) revealed strong expressions of α-SMA. These findings indicated high expression of α-SMA in adenomatoid odontogenic tumors.

**Figure 2:**
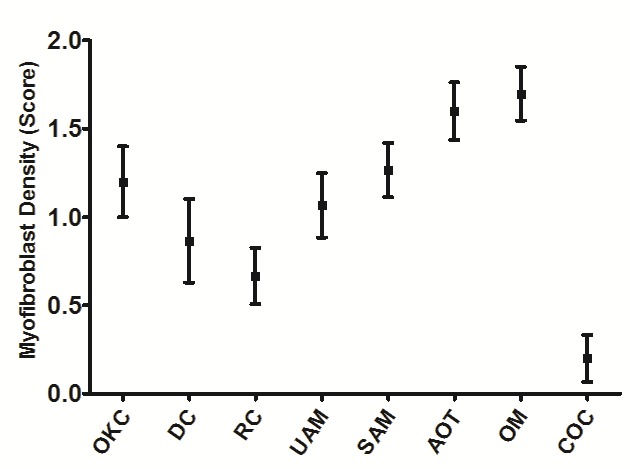
Error-bar graph of myofibroblast density in odontogenic cysts and tumors. OKC: odontogenic keratocyst; DC: dentigerous cyst; RC: radicular cyst; UAM: unicystic ameloblastoma; SAM: solid ameloblastoma; AOT: adenomatoid odontogenic tumor; OM: odontogenic myxoma; COC: calcifying odontogenic cyst.

### Odontogenic myxoma

None of the 10 odontogenic myxoma cases revealed negative expression of α-SMA. Three specimens (30%) had poor expression and 7 specimens (70%) had strong expression, indicating high expression of α-SMA in odontogenic myxoma.

Data from immunohistochemical analyses are summarized in Table 1.

Statistical analysis of data did not reveal a significant difference in mean MF density between the study groups‏ (P < 0.001). The intensity of MFs was significantly higher in odontogenic tumors (including myxoma, AOT, unicystic ameloblastoma and solid ameloblastoma) compared to odontogenic cysts (including DC, COC, OKC, and RC) (P < 0.001). There was no statistically significant difference between odontogenic tumors, except between UAM and OM (P = 0.041). The difference between OKC and odontogenic tumors was not statistically significant (P > 0.05). The number of MFs was significantly higher in OKC and lower in COC compared to other odontogenic cysts (P = 0.007 and P = 0.045, respectively). α-SMA expression in odontogenic lesions is illustrated in Figure 2.

## Discussion

Both odontogenic cysts and tumors originate from odontogenic epithelium.^1,2^ However, these lesions show different degrees of aggressiveness and biological behavior. A wide range of epithelium-associated factors have been indicated in the biological behavior of odontogenic lesions, including increased expressions of various proliferative markers such as ki-67, impaired expression of tumor suppressor genes and their products, and abnormal cell-cycle pathways.^3,12,13^ Only a few studies have assessed non-epithelial factors that could participate in the variable biological behaviors of different types of odontogenic cysts and tumors.^5,9^ Presence of MFs in the stroma plays an important role because of the ability of these cells to produce extracellular matrix components such as collagen and angiogenic factors, and synthesize enzymes and proteinases such as MMP-2, that can affect the tumor growth and progression.^3,11,14^

Shimasaki et al^15^ evaluated the distribution of MFs in bladder carcinoma and its relation with tumor invasiveness. The results of this study showed an increase in the number of α-SMA-positive cells during carcinogenesis, which suggests the role of these cells in tumor invasive characteristics. The direct relation between myofibroblasts’ density and invasive tumor characteristics has also been suggested by Tuxhorn et al^16^ in human prostate cancer. Seifi et al^17^ in the immunohistochemical evaluation of MFs in oral squamous cell carcinoma (SCC), oral epithelial dysplasia and hyperkeratosis showed that the number of α-SMA-positive MFs increased during carcinogenesis, which may support the role of MFs in tumor invasiveness.

In the present study, the mean MF densities in odontogenic cysts were as follows in descending order: OKC, DC, RC and COC. The expression of MFs in radicular cysts (RC), dentigerous cysts (DC) and odontogenic keratocysts (OKC) was analyzed by Nadalin et al.^11^ They reported that α-SMA-positive cell counts increased in OKC, followed by DC and RC, which was consistent with our findings. Furthermore, our results showed that α-SMA expression decreased in COC compared to various odontogenic cysts.

In the present study, the mean MF densities in odontogenic tumors were as follows in descending order: OM, AOT, SAM and UAM. Reddy et al^7^ in a study on follicular ameloblastomas, Pindborg tumors, ameloblastic fibro-odontomas, adenomatoid odontogenic tumors and odontogenic myxomas (with small sample sizes) found limited expression of MFs in follicular ameloblastoma and ameloblastic fibro-odontoma, which was restricted just around the blood vessels, while medium densities of MFs were seen in AOT and pindborg tumor, and high density of MFs was observed in odontogenic myxoma (in the population of spindle and stellate cells). They concluded that the increased density of MFs was associated with aggressive biological behavior of odontogenic tumors. The results of the study above in the cases of AOT and myxoma are consistent with our findings. However, in the case of follicular ameloblastoma (the most common form of solid ameloblastoma), which is an aggressive tumor, the results are not consistent. According to our observation, islands of odontogenic epithelium as well as the periphery of blood vessels were surrounded by α-SMA + cells, and relatively high densities of MFs were seen in solid ameloblastoma.

Based on our findings, α-SMA expression in OKC was very similar to that in more aggressive lesions (odontogenic tumors) compared to the expression of this protein in DC, RC and COC. These findings support the theory of the classification of OKC in odontogenic tumors category. In a study performed by Mashhadiabbas et al,^5^ there were no significant differences between MF densities in OKC and DC and in ameloblastoma and DC, while in OKC this was significantly higher than ameloblastoma. α-SMA-positive cells in DC and OKC were distributed in the subepithelial region and through the cyst wall. In the cases of ameloblastoma, myofibroblasts were located in the connective tissue close to the islands and in the stroma far from the islands. They concluded that the presence of MF in the stroma of the odontogenic lesions had no relationship with aggressive behavior.

The expression of α-SMA in solid ameloblastoma was significantly higher than unicystic ameloblastoma in this study. Vered et al,^3^ in a study on MFs among cases of DC, OKC, orthokeratinized odontogenic cyst, ameloblastic fibroma/fibro-odontoma, unicystic ameloblastoma, and solid ameloblastoma (with relatively small sample sizes), showed that the mean number of MFs in solid ameloblastoma was more than that in unicystic ameloblastoma, which is consistent with our findings. In the mentioned study, non-aggressive lesions showed lower expression of α-SMA compared to aggressive lesions. α-SMA-positive cells were mostly located parallel to and beneath the basement membrane of the odontogenic epithelium in the cystic lesions. Islands of odontogenic epithelium, especially in SAM, were surrounded by myofibroblasts.

## Conclusion

The results of the present study showed a significant role for MFs in aggressive behavior of odontogenic lesions and we suggest MFs as an important target for nonsurgical treatments. Realizing that MFs are a part of the stroma contributing to the progression of these lesions, future therapies may target stromal constituents and should not focus solely on conventional concepts. In this work, we attempted to accomplish a comprehensive study on a wide spectrum of odontogenic lesions (four odontogenic cysts and five odontogenic tumors) with a large sample size. However, we recommend further investigations to confirm the potential prognostic value of MFs in aggressive behavior of each odontogenic lesion, particularly solid ameloblastomas (an area of most conflict in previous studies). This may help predict the behavior of the lesions at the time of initial biopsy, which would be a guide for treatment strategies.

## Acknowledgements

This paper was written based on an undergraduate thesis (No. 1442) registered at Tabriz University of Medical Sciences, Faculty of Dentistry.

## Authors’ contributions

MK participated in study design, literature search, data acquisition, data analysis and manuscript preparation. MK participated in literature search, data acquisition, data analysis and manuscript preparation. GJ performed the paraclinical studies and participated in data acquisition and manuscript preparation. All the authors have read and approved the final manuscript.

## Funding

This study was financially supported by Tabriz University of Medical Sciences.

## Competing interests

The authors declare that they have no competing interests with regards to authorship and/or publication of this paper.

## Ethics approval

The research protocol was approved by the Ethics Committee of Tabriz University of Medical Sciences (Ref. No. 450).
